# *Aedes aegypti* Males as Vehicles for Insecticide Delivery

**DOI:** 10.3390/insects10080230

**Published:** 2019-08-01

**Authors:** Corey L. Brelsfoard, James W. Mains, Steve Mulligan, Anthony Cornel, Jodi Holeman, Susanne Kluh, Andrea Leal, Lawrence J. Hribar, Harold Morales, Tanya Posey, Stephen L. Dobson

**Affiliations:** 1 MosquitoMate, Inc., 2520 Regency Rd., Lexington, KY 40503, USA; 2 Consolidated Mosquito Abatement District, 2425 Floral Ave., Selma, CA 93662, USA; 3 Department of Entomology and Nematology, University of California, Davis; Davis, CA 95616, USA; 4 Greater Los Angeles Vector Control District, 12545 Florence Ave., Santa Fe Springs, CA 90670, USA; 5 Florida Keys Mosquito Control District, 18 Aquamarine Drive, Key West, FL 33040, USA; 6 Entomology Department, University of Kentucky, Lexington, KY 40546, USA

**Keywords:** autodissemination, pyriproxyfen, mosquito control

## Abstract

*Aedes aegypti* continues to spread globally and remains a challenge to control, in part due to its ‘cryptic behavior’ in that it often deposits eggs (oviposits) in larval habitats that are difficult to find and treat using traditional methods. Auto-dissemination strategies target these cryptic breeding sites by employing mosquitoes to deliver lethal doses of insecticide. This report describes the initial field trials of an application known as Autodissemination Augmented by Males (ADAM), utilizing *A. aegypti* males dusted with pyriproxyfen (PPF). Findings presented here are drawn from both caged and field trial studies. Together, these trials examined for the ability of *A. aegypti* males to disseminate PPF and to impact field populations. PPF-dusted males were able to effectively deliver lethal doses of PPF to oviposition sites under the conditions tested. Results from field trials in Florida and California demonstrated reduced *A. aegypti* populations in treated areas, compared to areas where PPF-treated males were not released. These results indicate that the release of PPF-dusted *A. aegypti* males can impact *A. aegypti* populations as measured by both reduced larval survival and lower numbers of adult female *A. aegypti*. We propose the ADAM approach as an addition to existing mosquito control techniques targeting *A. aegypti* and other mosquitoes that utilize cryptic larval habitats.

## 1. Introduction

Mosquito-borne diseases continue to impact the health of populations of many of the world’s tropical and subtropical regions. In the absence of approved vaccines, therapeutics, or prophylaxis, mosquito control remains the primary tool for combating many of these diseases, including dengue, chikungunya, and Zika. These viruses are often transmitted by *Aedes aegypti*, and the establishment of *A. aegypti* in an area increases the risk of autochthonous disease transmission [1].

As *A. aegypti* continues to spread, so does the risk of vector-borne disease transmission. In North America, *A. aegypti* primarily occurs in the southern United States, from Arizona eastward and below 33° N latitudes [2]. In 2017, the continental U.S. reported 5102 symptomatic Zika virus disease cases. Of those, 224 are attributed to local mosquito-borne transmission. The U.S. territories reported an additional 36,079 cases, including 35,937 attributed to local mosquito-borne transmission [3]. As *A. aegypti* spreads, the need for continued exploration of potential control strategies grows. 

*Aedes aegypti* often oviposit in artificial water containers associated with human activity—for example, flower pots, buckets, gutters, etc. Finding and treating these containers, often referred to as “cryptic breeding sites,” can present a challenge [4,5,6,7,8]. Successfully reducing adult *A. aegypti* populations, however, requires adequate coverage of cryptic breeding sites [9,10,11,12,13]. Auto-dissemination strategies target cryptic breeding sites by relying on mosquitoes themselves to deliver lethal doses of insecticide [14,15,16,17]. Auto-dissemination methods often employ insect growth regulators such as methoprene or pyriproxyfen (PPF), an active ingredient that is neither lethal nor repellant to adult mosquitoes [16]. Instead, methoprene or PPF acts as a potent inhibitor of immature mosquito development. PPF is an ideal candidate for autodissemination because it remains lethal at concentrations (LC50) of >0.012 parts per billion [15]. Approximately 1/1000 th the dry weight of a mosquito adult would adequately treat a 200 mL breeding site [18]. Residual activity of PPF can persist for up to four months [14]. Moreover, *Aedes* mosquitoes have demonstrated no resistance to PPF to date [19]. PPF, a juvenile hormone analogue, is not harmful to vertebrates. The WHO has, in fact, defined safe levels for direct treatment of drinking water [20]. 

The present study evaluates a technique called Auto-Dissemination Augmented by Males (ADAM). The ADAM approach involves the release of laboratory-reared males, which have been dusted with PPF into an infested area. A prior field study using ADAM has demonstrated *Aedes albopictus* males to be effective in transferring lethal PPF concentrations both to breeding sites and to conspecific female mosquitoes [17]. Using ADAM, the present study similarly examines the effectiveness of *A. aegypti* males at disseminating PPF to larval breeding sites. Specifically, hypotheses include that an appropriate dosage of PPF applied to adult *A. aegypti* males would not result in high levels of acute mortality, male quality would not be impacted from shipment of PPF treated males from a remote production facility to field release locations, that released PPF-dusted males would deliver PPF into introduced containers, and that the adult *A. aegypti* population would be significantly reduced following the release of PPF-dusted males, relative to the *A. aegypti* population at a similar, untreated area.

This study included both caged- and open-release trials. In cage studies, the effect of PPF dusting on *A. aegypti* male survivorship and the ability of PPF dusted *A. aegypti* males to deliver lethal doses to artificial oviposition containers were assessed. In the open-release trials, PPF-treated males were introduced into *A. aegypti* infested sites in Clovis, CA; Key Largo, FL; and Los Angeles, CA. These open releases utilized male *A. aegypti,* which were produced and PPF-dusted at a rearing facility in Kentucky and then shipped to release locations. Measurements assessed shipment survivorship as well as *A. aegypti* male’s ability to successfully transfer PPF to artificial oviposition sites. The results demonstrate that PPF-dusted adult *A. aegypti* males (1) can be shipped cross-country with little effect on survivorship and (2) can deliver lethal doses of PPF to artificial oviposition sites in a field setting.

## 2. Materials and Methods

*Aedes aegypti* mosquitoes used in caged experiments, bioassays, and field releases in Los Angeles and Florida were the WACO strain [21]. *Aedes aegypti* mosquitoes used for Clovis shipments and release were from a colony derived from Clovis, California [22]. Larvae were fed with a 60 g/L liver powder (ICN Biomedicals, Irvine, CA, USA) slurry. Adults were held in 24.5 cm × 24.5 cm × 24.5 cm BugDorm-4S2222 insect rearing cages (MegaView Science Co., Taichung, Taiwan) and provided a constant supply of 10% sucrose. Females were provided with bovine blood for egg production. All experiments used adult young male mosquitoes that were less than 2 d post emergence at the time of release. Prior to the experiments described here, multiple dose-response treatments were performed to determine a dusting rate that did not appear to affect adult *A. aegypti* male survival [17]. For all experiments, the PPF treatment consisted of a 30%:70% mixture of Esteem 35WP (Valent Biosciences, Libertyville, IL, USA) and DayGlo fluorescent powder (DayGlo Color Corp., Cleveland, OH, USA), respectively. Mosquitoes were dusted using a hand bellow powder duster (Harris Manufacturing Co. LLC, Cartersville, NC, USA) in cardboard mailing tubes (63.5 mm diameter 20.3 cm long), capped on both ends with No-See-Um netting (Equinox, Williamsport, PA, USA). Maps of field sites were generated using OpenStreetMap^®^ (https://www.openstreetmap.org).

### 2.1. Laboratory Survivorship and Mesocosm Cage Studies

The study first examined for effects of PPF treatment on adult survivorship. Males were treated with PPF as described in the previous section. Two replicates of 30 *A. aegypti* males were dusted with PPF and released into a cage (30 cm × 30 cm × 30 cm). For comparison, two additional replicates of 30 males were left undusted, and survivorship was then monitored in all four cages.

A second study examined for the transfer of PPF directly from males into oviposition containers. To exclude the possibility of indirect PPF transfer to breeding sites, i.e., females delivering PPF into the water after having become PPF contaminated by males, no females were introduced into the mesocosm cage for this experiment. Two hundred and fifty dusted *A. aegypti* males were released into a mesocosm cage (L × W × H; 4 m × 3.5 m × 2.2 m) containing five oviposition containers (16 oz. Plastic Container, 128HRD16 COMBO240, webstaurant.com) containing 100 mL water and lined with seed germination paper (Anchor Paper Company, St. Paul, Minnesota). As a control, 250 untreated males were released into a second, identical mesocosm cage. Both mesocosm cages were housed in a climate-controlled greenhouse (25.5 ± 2.9 °C; RH 74.2 ± 4.1%). Five days after mosquito introduction, all mosquitoes were removed from each of the mesocosm cages. At the same time, the oviposition containers were also removed, and 25 mL samples of water from each were examined using the Kentucky (KY) bioassay method: For the KY bioassay, three samples were placed in 60 mL plastic cups (Dart, Mason, Michigan) with four L3 larvae and three drops of a 60 g/L liver powder slurry. Bioassays measured the number of emerging adults and dead immatures in each cup.

A third experiment assessed the indirect transfer of PPF from males to females. In this experiment, 250 dusted males were placed into the mesocosm cage along with 100 undusted females. As a control, 250 undusted males were released into an identical mesocosm cage along with 100 undusted females. After four days, the females were individually aspirated using different aspirator tips to avoid contamination from both cages and individualized into separate containers to minimize any subsequent transfer of PPF after removal from cages. To assess for PPF on the undusted females, each female was individually examined using the above-described KY lab bioassay method.

### 2.2. Mosquito Shipment

**Figure 1:**
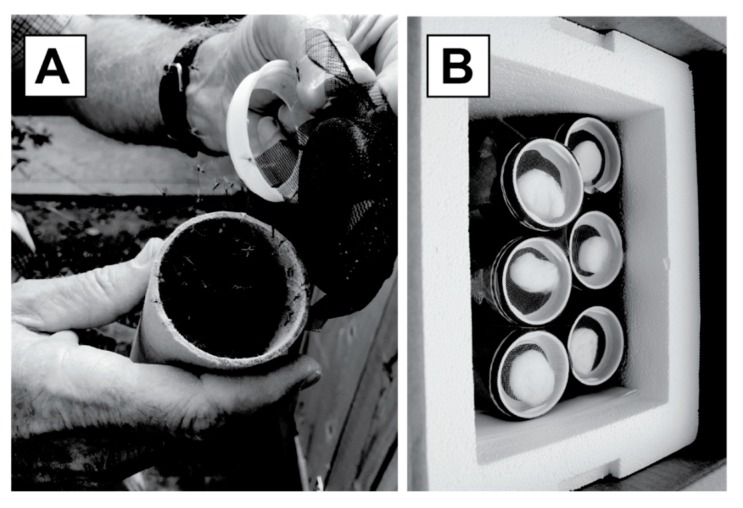
(**A**) Release of *Aedes aegypti* PPF-treated males from cardboard shipping tubes. (**B**) Shipping container with cardboard tubes topped with mesh and a sucrose-soaked cotton ball.

Open release trials utilized successful shipments of *A. aegypti* males that were produced and dusted in Kentucky. The males were transported via commercial courier in cardboard mailing tubes. As a source of water and food during shipment, cotton balls soaked with 10% sucrose solution were placed at both ends of each tube (Figure 1). One thousand adult male *A. aegypti* mosquitoes were anesthetized with chloroform (Fisher Scientific), counted, and placed in each tube. For each shipment, tubes were positioned in a 17.5 cm × 22.5 cm × 30 cm Styrofoam cooler, along with a water-moistened paper towel and a Hobo sensor to monitor conditions (Onset Computer Corporation, Bourne, Massachusetts; Figure 1). The sensors tracked temperature, light, and humidity during transit for all shipments, with one exception, where a temperature sensor malfunctioned. Coolers were shipped overnight, and in no shipment did the mosquitoes remain in the package for more than 24 hours [23]. In total, nine shipments were made, with 5–6 tubes each. The shipment process allowed for measurement of mosquito survivorship during cross-country delivery by the commercial courier. After the mosquitoes were released from tubes in California, the number of dead males remaining in the tubes was counted and recorded. 

### 2.3. Fresno/Clovis, CA Field Releases

**Figure 2:**
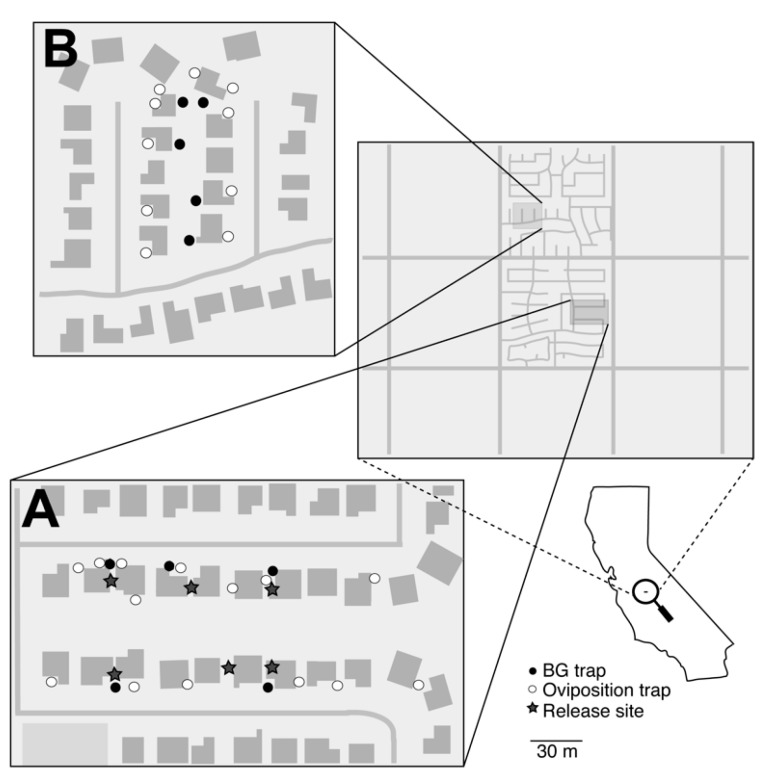
Map of the (**A**) treated and (**B**) untreated locations in Clovis, CA. Open circles represent artificial oviposition sites that were surveyed for eggs, larvae, and the presence of PPF using bioassays. Closed circles represent BG traps used to monitor adult populations. The stars represent release locations of PPF-treated *A. aegypti* males.

Clovis, CA has an arid climate with little rainfall (~311 mm annually) throughout the year. The *A. aegypti* populations targeted in Clovis were recently established and still expanding at the time of the trial [24]. The treated and untreated areas in Clovis, CA together encompassed approximately five acres of urban residential neighborhood (Figure 2). Releases at the treated area were approved by the California Department of Pesticide Regulation [25]. To raise local awareness about the project, the Consolidated Mosquito Control District produced an educational online video (http://mosquitobuzz.net/AedesaegyptiADAM.htm) and distributed informational flyers prior to the trial. In addition, several local media outlets produced articles and short news segments intended to inform the public. 

Prior to the release, 14 and 10 artificial oviposition sites (ovisites) were placed at the treated and untreated areas, respectively. Each ovisite consisted of a 24-ounce glass mason jar (Jarden Home Brands) painted with black enamel paint (Rust-Oleum Inc.) and held in place with a metal tent peg (Essentials Tool Inc.). Five Biogents sentinel (BG) traps (Biogents AG, Regensburg, Germany) were baited with CO_2_ (dry ice, 1–1.5 kg) and a BG lure (Biogents AG, Regensburg, Germany) and placed within each of the treated and untreated areas (Figure 2). Ovisites were placed in either the front or back yard of residents’ homes, out of direct sunlight and away from yard irrigation. Cups (16 oz. Black Stadium Cups, Promotion Choice) were lined with germination paper (400PT Non-Toxic Paper Toweling; Seedburo) and placed in small ceramic pots to prevent them from tipping over. Finally, to avoid disturbances from animals, large gauge mesh netting (DeerBlock, Easy Gardener Inc., Batesburg, SC, USA) was fitted over the top of each.

Groups of approximately 5000 PPF dusted males were released within the treatment area twice per week for a total of five weeks. Males were released by hand from the tubes near vegetation at multiple locations within the field site (Figure 2). Typically, males were released immediately following their arrival by overnight transit to the release locations. Since the commercial shipment timing, male releases were typically performed in the early afternoon (i.e., noon–5pm). Due to repeated releases and the potential for transfer of dust to collected wild-type mosquitoes, including the potential for dust transfer occurring in trap nets, our experimental design did not include monitoring for ‘recaptured males,’ i.e., we did not perform a mark release recapture (MRR) type experiment due to potential artifacts caused by dust accumulation from repeated releases across multiple weeks. Adult populations were monitored weekly with BG traps that were baited with dry ice and a BG lure and were deployed for a single trap night (~24 hours). Ovisites were sampled weekly by transferring approximately 30 mL of water from each ovisite into a 60 ml plastic cup (Dart, Mason, Michigan). The seed germination paper was also removed each week, and cups were relined with new paper and refilled with tap water. Ovisite water samples were bioassayed in the lab using the California method, which consisted of adding four L3 larvae and 0.01g of ground TetraMin^®^ (Tetra, Spectrum Brands Pet, LLC, Blacksburg, VA, USA) (tropical flakes fish food) to the 30 mL of water collected from each ovisite. Bioassays recorded the number of emerging adults and dead immatures in each cup.

### 2.4. Florida Keys, FL and Los Angeles, CA Field Releases

The climate of the Florida Keys, FL is tropical with an annual precipitation rate of 1267 mm of rainfall per year. *A. aegypti* populations with the Florida Keys are well established and can pose a significant health threat to local residents. Los Angles has a warm and temperate climate with rainy and dry seasons. As with Clovis, CA, the *A. aegypti* populations targeted in Los Angeles were recently established and still expanding at the time of the trial [24]. 

**Figure 3:**
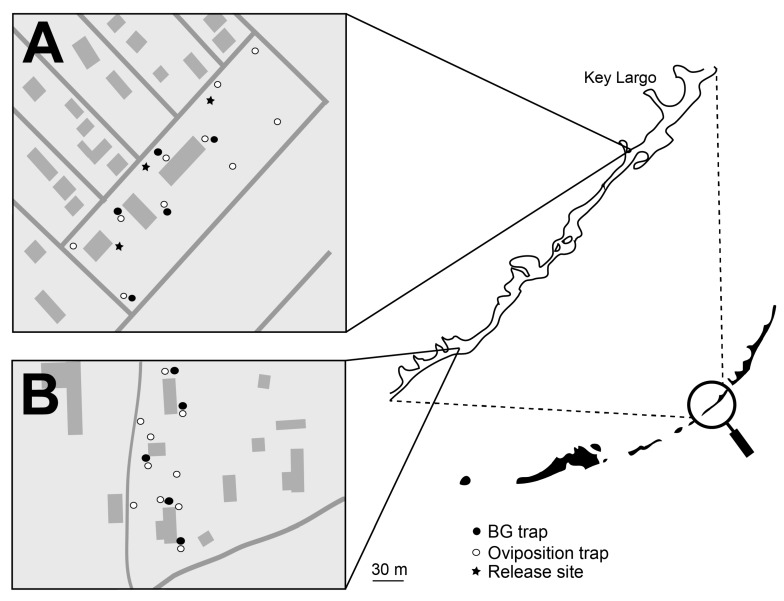
Map of the (**A**) treated and (**B**) untreated locations in the Florida Keys, FL. Open circles represent artificial oviposition sites that were surveyed for eggs, larvae, and the presence of PPF using bioassays. Closed circles represent BG traps used to monitor adult populations. The stars represent release locations of PPF-treated *A. aegypti* males.

**Figure 4:**
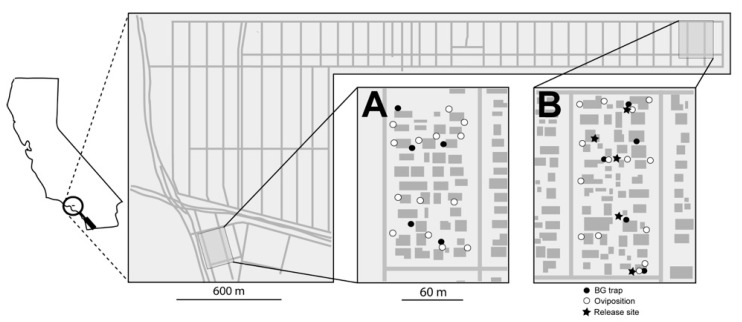
Map of the (**A**) untreated and (**B**) treated locations in Los Angeles, CA. Open circles represent artificial oviposition sites that were surveyed for eggs, larvae, and the presence of PPF using bioassays. Closed circles represent BG traps used to monitor adult populations. The stars represent release locations of PPF-treated *A. aegypti* males.

Treated and untreated areas were defined in the Florida Keys (Figure 3) and in Los Angeles, CA (Figure 4). Each area included approximately five and eight acres in Florida and Los Angeles, respectively. Ten ovisites and five BG Sentinel traps were placed at each area. In FL, BG traps were baited with dry ice and a BG lure and were set for ~24 hours, similar to the procedure utilized in Clovis, as described above. In Los Angeles, BG traps were baited with BG lures only (no CO_2_). Ovisites were made from 700 mL glass jars (Ball Corporation, Broomfield, CO, USA) that were painted black externally using Flexseal (Flex Seal, Dayton, NJ, USA). These were filled with water, positioned near vegetation and lined with seed germination paper, which was removed and replaced weekly.

PPF-dusted *A. aegypti* males were released in an area within Los Angeles, CA, across an eight-week period. Male release methodology was analogous to that used in Clovis, CA. Twice per week, approximately 5000 PPF-dusted males were released at the treated area (i.e., total of 10,000 males/week) near vegetation at multiple locations within the field site. Ovisite water samples were collected weekly, and bioassays were performed using a modification on the KY bioassay method. Specifically, the standard method was followed, except samples were placed in 20 mL borosilicate glass scintillation vials (Kimble, Vineland, NJ, USA) with four L3 larvae and three drops of a 60 g/L liver powder slurry. In addition to monitoring during the eight-week release period, monitoring was also conducted during a two-week pre-release period and a two-week post-release period.

Concurrent with the Los Angeles study, PPF-dusted *A. aegypti* males were released at an area on Key Largo, FL. Male release methodology was analogous to that used in Clovis, CA. The overall design was similar to the Los Angeles study: Bioassays were performed with water samples from the treated and untreated areas, and male and female adult *A. aegypti* were monitored using BG traps. As with the Clovis study, male recapture assessments using the fluorescent dust were not part of the study design due to the potential transfer of dust between marked and unmarked specimens and artifacts from multiple releases of dusted males within the treated area. The two areas were monitored for a four-week pre-release period, a six-week release period, and a two-week post-release period. In the Key Largo study during the post-release period, approximately half of the traps were disrupted due to weather events.

### 2.5. Statistical Analyses

Statistical analyses were performed using JMP 12.1 (SAS Institute, Cary, NC, USA). Log-rank tests were used to compare mortality of treated and untreated males over time. ANOVA was used to examine for differences in dusted male survival, comparing between shipments; proportional survivorship data were arcsine transformed. Subsequently, bivariate correlations were used to examine for effects of temperature on male survival during shipment. Contingency analysis was used to compare the number of surviving adults in bioassays, and the Wilcoxon exact test was used to examine for differences in adult female and male number at field sites. 

## 3. Results

### 3.1. Laboratory Survivorship and Mesocosm Cage Studies

In laboratory cage experiments comparing survivorship of adult males, neither group was observed to experience acute mortality, with a 20 day median survival time of treated males. The treated group was observed to have a decreased survivorship compared to the untreated control group (log-rank, *X*^2^ = 25.1, *p* < 0.0001), which had a median survival of 31 days.

In the mesocosm studies, results show that PPF-treated males can directly deliver PPF into the ovisite water sources in the absence of females. Water samples from the treated mesocosm cage were significantly (*X*^2^ (4, *N* = 40) = 21.85, *p* < 0.0002) more toxic to larvae in bioassays, relative to water samples removed from the untreated cage. Specifically, in bioassays of water samples taken from the treated mesocosm cage, the observed larval survivorship was significantly lower (1.9 ± 0.3 eclosed adults; mean ± SE) than that observed in bioassays of samples taken from the untreated cage (3.7 ± 0.2 eclosed adults). 

In a third experiment, the mesocosm cages were used to examine for transfer of PPF from males to females. Consistent with the hypothesized transfer of PPF from males to females, increased larval mortality (*X*^2^ (4, *N* = 24) = 27.86, *p* < 0.0001) was observed in bioassays that included females removed from treated cages (0.9 ± 0.3 eclosed adults), compared to females removed from the untreated cage (3.6 ± 0.1 eclosed adults). Similar results were observed with males removed from the treated cages (0.8 ± 0.3 eclosed adults), compared to males removed from the untreated cage (3.8 ± 0.1 eclosed adults; *X*^2^ (3, *N* = 24) = 27.86, *p* < 0.0001). Water samples removed from ovisites also revealed significantly different bioassay mortality (*X*^2^ (4, *N* = 40) = 21.63, *p* < 0.0002) between the treated cage (2.1 ± 0.3 eclosed adults) and the untreated cage (3.6 ± 0.1 eclosed adults). 

### 3.2. Survivorship of PPF Dusted A. aegypti Males During Shipment to Clovis, CA

**Figure 5:**
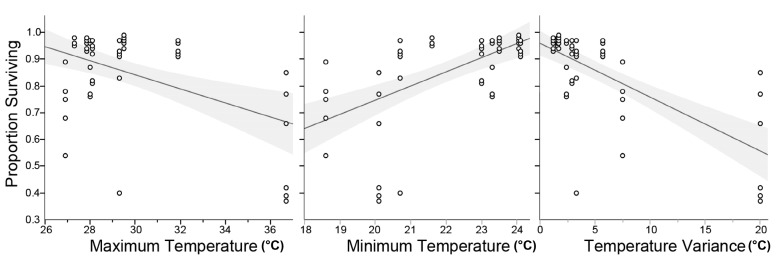
Survivorship of PPF-treated males shipped from Lexington, KY to Fresno, CA: Maximum, minimum, and temperature variance.

Mean survivorship for all shipments was 86% ± 2% (Mean ± SE). Mortality varied among treatments, i.e., shipments (*F*(8,43) = 9.62, *p* < 0.0001). Since the internal temperatures of shipping packages varied, we used bivariate correlation to examine for a relationship between mortality and temperatures experienced during shipment. As shown in Figure 5, survival was negatively correlated with both the maximum (*r*(52) = 0.22, *p* < 0.0001) and minimum (*r*(52) = 0.35, *p* < 0.0001) temperatures. Greater mortality was also observed with males that experienced greater temperature variations during shipment (*r*(52) = 0.48, *p* < 0.0001). 

### 3.3. Field Releases of PPF-Treated Males—Clovis, CA

**Figure 6:**
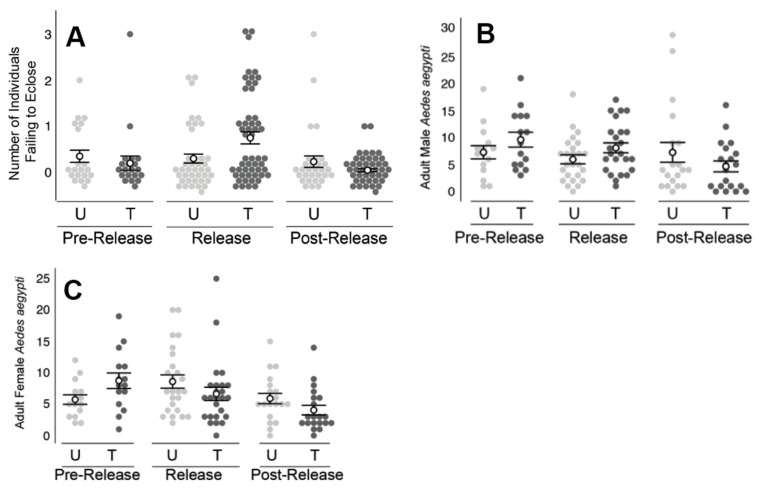
Results from field releases in Clovis, CA. (**A**) During the release period only, individuals reared in water samples from the treated (T) area experienced higher mortality (*p* < 0.05), relative to that observed from the untreated (U) area. (**B**) During the release period only, the number of adult males in the treated area was slightly elevated (*p* < 0.05) relative to that observed in the untreated area. (**C**) Between the pre-release and release periods, the number of adult female *A. aegypti* decreased at the treated area. In contrast, the number of *A. aegypti* females at the untreated area was observed to increase during the same time periods. Filled circles represent the raw data measurements. Hollow circles are the means with standard error bars.

In bioassays performed with water samples drawn from ovisites, a significant difference (*X*^2^ (3, *N* = 92) = 7.92, *p* < 0.05) was observed between the treated and untreated areas only during the release period (Figure 6a). 

During the five-week release period, no difference (*Z* = −1.59, *p* > 0.11) was observed in the number of adult *A. aegypti* males (8.1 ± 0.9 males/collection) collected in BG traps relative to that of the untreated area (6.0 ± 0.8 males/collection; Figure 6b). Similarly, during the three-week pre-release and four-week post-release periods, comparisons of adult male number showed no significant difference between the two areas (*p* > 0.27). 

The initial number of female *A. aegypti* collected in BG traps was slightly higher (*Z* = −1.87, *p* > 0.058) at the treated site (8.7 ± 1.2 females/collection) during the pre-release period, compared to the untreated area (5.7 ± 0.8 females/collection). Following the start of male releases, the number of females was not observed to differ significantly (*p* > 0.8) between the treated and untreated areas during either of the release or post-release periods (Figure 6c).

### 3.4. Field Releases of PPF-Treated Males—Los Angeles, CA

**Figure 7:**
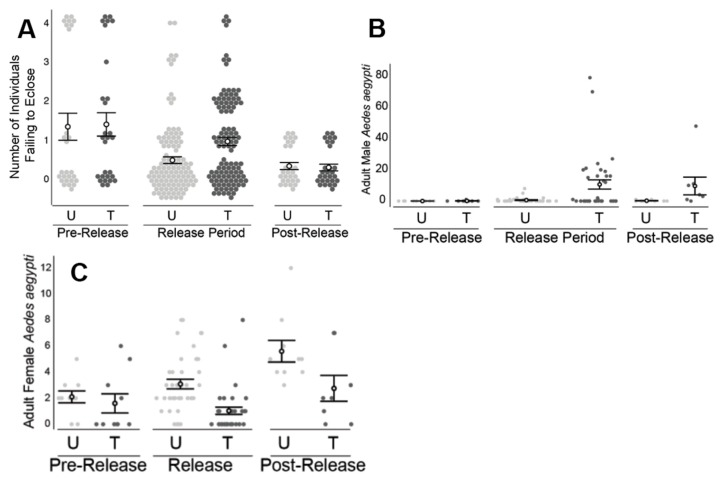
Results from field releases in Los Angeles, CA. (**A**) During the release period only, individuals reared in water samples from the treated (T) area experienced higher mortality (*p* < 0.0001), relative to that observed from the untreated (U) area. (**B**) During the release period only, the number of adult males in the treated area was elevated (*p* < 0.005) relative to that observed in the untreated area. (**C**) During the release (*p* < 0.0001) and post-release (*p* < 0.05) periods, a lower number of adult females was observed in the treated area relative to that observed in the untreated area. Filled circles represent the raw data measurements. Hollow circles are the means with standard error bars.

During the release period, larvae exposed to water from the treated area experienced greater mortality in bioassays when exposed to water from the treated area (*X*^2^ (4, *N* = 222) = 25.534, *p* < 0.0001) compared to larvae exposed to water from the untreated area (Figure 7A). In contrast, during both the pre-release and post-release periods, comparisons of water bioassays showed no significant difference between the treated and untreated areas.

During the pre-release period, no significant difference was observed in the number of adult male *A. aegypti*, comparing the treated and untreated areas. Only one male was collected across both areas (Figure 7B). During the release period, the number of males collected at the untreated area remained low (0.6 ± 1.6 males/collection). In contrast, during the release period, the number of males collected in the treated area increased (10.6 ± 18.0 males/collection) and was significantly higher than the number collected in the untreated area (*Z* = 2.96, *p* < 0.0027). During the post-release period, the male number in the treated area remained elevated (9.6 ± 16.1 males/collection) relative to that of the untreated area (0.2 ± 0.4 males/collection; *Z* = 2.59, *p* < 0.006).

The female *A. aegypti* number was not observed to differ significantly between the treated (1.6 ± 0.7 females/collection) and untreated (2.1 ± 0.5 females/collection) areas during the pre-release period (Figure 7c). During the release period however, significantly fewer (*Z* = −4.63, *p* < 0.0001) *A. aegypti* females were collected at the treated area (1.0 ± 0.3 females/collection) relative to the untreated area (3.1 ± 0.4 females/collection) resulting in an estimated 66% reduction in the female population. During the two-week post-release period, the number of females remained lower (*Z* = −2.16, *p* < 0.035) at the treated area (2.8 ± 1.0 females/collection) compared to the untreated area (5.6 ± 0.8 females/collection).

### 3.5. Field Releases of PPF-Treated Males—Florida Keys

**Figure 8:**
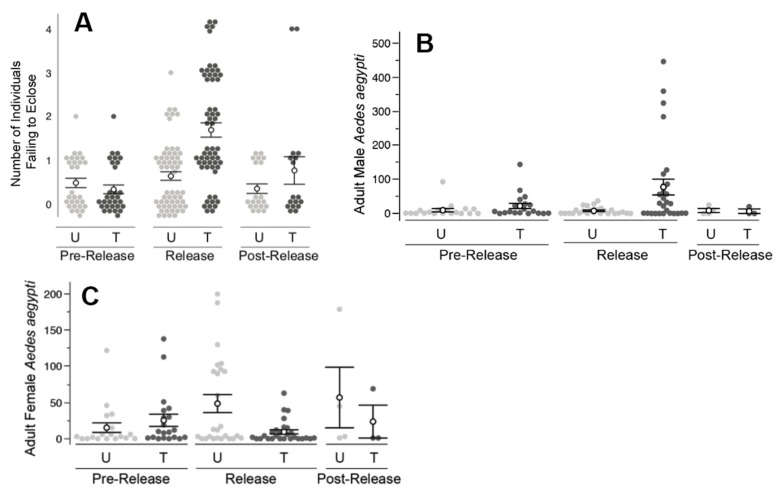
Results from field releases in the Florida Keys. (**A**) During the release period only, individuals reared in water samples from the treated (T) area experienced higher mortality (*p* < 0.0001), relative to that observed from the untreated (U) area. (**B**) During the release period only, the number of adult males in the treated area was elevated (*p* < 0.01) relative to that observed in the untreated area. (**C**) During the release period only, a lower number of adult females was observed in the treated area relative to that observed in the untreated area (*p* < 0.01). Filled circles represent the raw data measurements. Hollow circles are the means with standard error bars.

During the release period, larvae experienced greater mortality in bioassays when exposed to water from the treated area (*X*^2^ (4, *N* = 116) = 29.43, *p* < 0.0001) compared to larvae exposed to water from the untreated area (Figure 8A). In contrast, during both the pre-release and post-release periods, comparisons of water bioassays showed no difference between the treated and untreated areas.

Overall, higher numbers of *A. aegypti* adults were observed in the Florida Keys, relative to either of the Clovis and Los Angeles areas. During the pre-release period, no significant difference was observed in the number of adult male *A. aegypti*, comparing the treated and untreated areas (Figure 8B). During the release period, the number of males collected at the untreated area remained low (7.8 ± 2.1 males/collection). In contrast, the number of males collected during the release period in the treated area increased (77.6 ± 23.1 males/collection) and was significantly higher than the untreated area (*Z* = −2.29, *p* = 0.021). During the post-release period, the male number declined in the treated area (6.7 ± 6.7 males/collection) and was not observed to differ significantly from that of the untreated area (8.8 ± 5.9 males/collection).

The female *A. aegypti* number was not observed to differ significantly between the treated (25.4 ± 8.5 females/collection) and untreated (15.3 ± 6.7 females/collection) areas during the pre-release period (Figure 7C). During the release period however, significantly fewer (*Z* = 1.92, *p* < 0.03) *A. aegypti* females were collected from the treated area (9.5 ± 2.9 females/collection) relative to the untreated area (48.6 ± 12.5 females/collection) resulting in an estimated 88% reduction in the female population. During the two-week post-release period, the number of females remained lower at the treated area (23.7 ± 22.7 females/collection) compared to the untreated area (57.0 ± 41.9), but the difference was non-significant.

## 4. Discussion

While previous work has demonstrated the ability of *A. albopictus* males to disseminate PPF [17], this is the first report of the ADAM method with *A. aegypti* populations. The primary goals of the work reported here were: 1) To determine the effects of PPF dusting on male survival, 2) to examine for an ability of ADAM dusted males to disseminate PPF both within artificial environments and in field trials, and 3) to examine for an impact of ADAM male releases on field populations of *A. aegypti* females.

Laboratory examination of adult male *A. aegypti* survival show slightly lower survival of PPF-dusted males, compared to undusted males. However, the effects were not acute, and males within both groups lived for greater than one month. While survival was not directly examined in the field, the results of field trials suggest that PPF-males do survive long enough to disseminate PPF in these conditions. ESTEEM 35WP is a wettable powder and that it includes ingredients (e.g., surfactants) that may be harmful to adult mosquitoes and, therefore, not ideal for the ADAM approach. Further work is needed to develop and test additional PPF formulations that may be less harmful to adult mosquitoes.

Female mosquitoes were intentionally excluded from the design of one mesocosm experiment reported here. This design was intended to examine for the direct dissemination of PPF by males. Male-dissemination in the absence of females would allow prophylactic treatment of potential larval sites before populations of female mosquitoes appear. As described previously [17,26,27,28], because artificially reared males can be produced at any time, the ADAM method is not necessarily reliant on naturally occurring mosquitoes. As a result, effective dissemination is not necessarily impacted by the rise and decline of the natural population. Unlike the prior *A. albopictus* study [17], the present study did not specifically examine for direct dissemination in the field, because female *A. aegypti* were present throughout the release periods. In other words, because males transfer PPF to females, the possibility cannot be excluded that all field dissemination occurred indirectly, due to male PPF transferal to female *A. aegypti*. To exclude this possibility, future studies might examine for dissemination early in the season, when female *A. aegypti* are rare or absent. This suggestion would be similar to the approach employed with *A. albopictus* in a previous report [17].

Immature mosquitoes exposed to water collected from ovisites near ADAM male releases experienced significantly higher mortality in all three field trials (Clovis, Los Angeles, and Florida Keys). However, the Clovis study was unlike the other two studies in that an obvious impact on the adult population was not observed. The Clovis study also varied from the other two studies in that, despite the repeated release of PPF-dusted males, BG trap collections detected only a slightly increased number of males, relative to the untreated areas. The number of adult *A. aegypti* females collected did not differ between the treated and untreated areas during the release period. It is of note, however, that the female number was significantly higher in the untreated area prior to the start of releases. As shown in Figure 6, between the pre-release and release periods, the number of female *A. aegypti* increased in the untreated area and decreased in the treated area, which suggests an ADAM effect on the *A. aegypti* population. While additional studies are needed, these results indicate that an important indicator of success is the detection of elevated male recapture rates. 

Similar to the Clovis work, field releases of PPF-dusted *A. aegypti* males in Los Angeles, CA and the Florida Keys also detected significantly higher mortality of immature mosquitoes exposed to water collected from ovisites near the releases of ADAM males, relative to water sampled from the untreated areas. The increased mortality was coincident with ADAM male releases and was not observed prior to the release of ADAM males nor immediately following the cessation of releases. The decrease in larval mortality following the cessation of releases may be due to the dilution of PPF in sample containers (e.g., due to rainfall and artificial watering of lawns). The types of containers sampled (i.e., small glass jars) were prone to drying out or wash out, particularly in the Clovis and Florida Keys field sites, respectively. Ovicups were often noted to be dry in Clovis when checked weekly. The observation of dry ovicups could also help explain the limited impact on the adult population, if dry ovicups were not attractive oviposition sites for *A. aegypti*.

In other ways, the results observed in Los Angeles and the Florida Keys adult collections differed from those observed in Clovis. Specifically, the male capture rates detected at both Los Angeles and the Florida Keys were much higher in the treated areas, and significant male increases were observed during the release period only. In other words, the treated and untreated pairs were not observed to differ in the number of *A. aegypti* females prior to the start of releases. Consistent with expectations for a population impact of ADAM males, a lower number of adult *A. aegypti* females was observed at the treated areas, and this decrease in the number of females was coincident with the start of male releases.

There are potential sources of variation that could have contributed to the observed results between the different field studies including: (1) The Clovis study was the first of the *A. aegypti* trials described here, (2) different cooperating personnel operated at each of the field locations, and (3) used differing methods (e.g., BG trapping in Los Angeles did not include the use of CO_2_), (4) the targeted populations of *A. aegypti* differed between the cities [29], (5) a different strain of *A. aegypti* was used in Clovis relative to the other two studies, and (6) the weather and geography of Clovis differs relative to the other two areas. Future examination could increase the number of males released in Clovis and examine for an elevated male recapture rate and impact on the female *A. aegypti* numbers. However, because fieldwork is currently constrained in scale by regulatory approvals, operating at a larger scale is not currently possible. At this small scale, immigration of females could have affected adult collection numbers, complicating interpretation of the experiment. Repeating the ADAM field studies in Clovis, Los Angeles, and the Florida Keys allowed us to examine the ADAM method in different environments, different types and densities of housing, and different climates, e.g., arid Clovis versus tropical Florida Keys. Furthermore, the overall *A. aegypti* population densities are higher in the Florida Keys, where *A. aegypti* have long been established, relative to Clovis and Los Angeles, where *A. aegypti* has only recently established [24]. Clearly, additional replication would be beneficial in assessing optimal male release rates, and the results reported here support the effectiveness of the ADAM approach for PPF dissemination across a range of habitats. Investigations into field performance of ADAM mosquito strains could be studied in upcoming field assessments. Male performance (i.e., male competitiveness) is widely considered a key factor in a successful sterile insect technique (SIT) programs, and PPF dissemination rates in the field could vary between mosquito strains and local environmental factors at a release location.

Ideally, the ADAM method would employ locally reared males, which would preclude the need for shipping adult *A. aegypti* males. However, the results presented here show that male *A. aegypti* can remain useful for PPF dissemination following cross country shipment. While the dusting and shipment affect male longevity, the results demonstrate that a sufficient number of males survive and disseminate PPF. Since the conditions during shipment (temperature extremes and variation) affect male fitness, additional work can investigate improved methods for shipment (e.g., how to better maintain a desired temperature during shipment), if future work cannot be based on local production of ADAM males.

Lastly, the fieldwork reported here took place in areas that were not co-infested with *A. albopictus* and *A. aegypti*. If possible, future studies might examine areas with both *A. albopictus* and *A. aegypti*. In these areas, the release of one species could be examined for an intra- and inter-specific effect on immature development. This is possible because both *A. albopictus* and *A. aegypti* larvae occur in similar habitats and because both are ‘container breeders’ [29]. Furthermore, *A. albopictus* males have been shown to attempt mating with *A. aegypti* females, known as ‘satyrization’ [30,31,32,33], which could result in the interspecific transfer of PPF from released *A. albopictus* males to *A. aegypti*. If this were demonstrated, then releases of one species could be used to control additional container-breeding mosquito species. The potential for interspecific effects also demonstrates the need to monitor for potential non-target impacts, e.g., non-Culicid insects that co-occur within the same containers.

## 5. Conclusions

Results here show that an ADAM approach based on the release of PPF-dusted *A. aegypti* males can impact *A. aegypti* populations as measured by both immature survival and numbers of adult female *A. aegypti*. Importantly, we do not envision the ADAM method as a stand-alone approach. Rather, we suggest its integration with existing mosquito control programs that also deploy traditional larviciding and adulticiding, e.g., manual- or machine-application of larvicides to large containers and ADAM to treat the small, cryptic containers. The integration of ADAM along with established mosquito control methods offers benefits. First, due to small PPF amounts and ‘mosquito-driven delivery’, the ADAM method will be more effective against relatively small containers and breeding sites where mosquito larvae develop. This is important because effective treatment of small, cryptic containers remains an important deficiency in many mosquito control programs [34,35]. Furthermore, the ADAM method can be overlaid onto existing autocidal methods (e.g., traditional sterile insect technique, incompatible insect technique, etc.) in which male mosquitoes are mass-produced and released. In conjunction with traditional methods, the ADAM approach could improve efficacy and affect interspecific larvae that co-infest the ADAM-treated areas. The potential benefit of the ADAM method to ‘boost’ autocidal approaches has been previously emphasized [17].
